# Utility of sequenced genomes for microsatellite marker development in non-model organisms: a case study of functionally important genes in nine-spined sticklebacks (*Pungitius pungitius*)

**DOI:** 10.1186/1471-2164-11-334

**Published:** 2010-05-27

**Authors:** Takahito Shikano, Jetty Ramadevi, Yukinori Shimada, Juha Merilä

**Affiliations:** 1 Ecological Genetics Research Unit, Department of Biosciences, University of Helsinki, P.O. Box 65, FI-00014, Helsinki, Finland; 2 School of Life Sciences, University of Hyderabad, Hyderabad 500 046, India

## Abstract

**Background:**

Identification of genes involved in adaptation and speciation by targeting specific genes of interest has become a plausible strategy also for non-model organisms. We investigated the potential utility of available sequenced fish genomes to develop microsatellite (cf. simple sequence repeat, SSR) markers for functionally important genes in nine-spined sticklebacks (*Pungitius pungitius*), as well as cross-species transferability of SSR primers from three-spined (*Gasterosteus aculeatus*) to nine-spined sticklebacks. In addition, we examined the patterns and degree of SSR conservation between these species using their aligned sequences.

**Results:**

Cross-species amplification success was lower for SSR markers located in or around functionally important genes (27 out of 158) than for those randomly derived from genomic (35 out of 101) and cDNA (35 out of 87) libraries. Polymorphism was observed at a large proportion (65%) of the cross-amplified loci independently of SSR type. To develop SSR markers for functionally important genes in nine-spined sticklebacks, SSR locations were surveyed in or around 67 target genes based on the three-spined stickleback genome and these regions were sequenced with primers designed from conserved sequences in sequenced fish genomes. Out of the 81 SSRs identified in the sequenced regions (44,084 bp), 57 exhibited the same motifs at the same locations as in the three-spined stickleback. Di- and trinucleotide SSRs appeared to be highly conserved whereas mononucleotide SSRs were less so. Species-specific primers were designed to amplify 58 SSRs using the sequences of nine-spined sticklebacks.

**Conclusions:**

Our results demonstrated that a large proportion of SSRs are conserved in the species that have diverged more than 10 million years ago. Therefore, the three-spined stickleback genome can be used to predict SSR locations in the nine-spined stickleback genome. While cross-species utility of SSR primers is limited due to low amplification success, SSR markers can be developed for target genes and genomic regions using our approach, which should be also applicable to other non-model organisms. The SSR markers developed in this study should be useful for identification of genes responsible for phenotypic variation and adaptive divergence of nine-spined stickleback populations, as well as for constructing comparative gene maps of nine-spined and three-spined sticklebacks.

## Background

Recent advances in our understanding of the physiological and molecular functions of genes have paved the road for investigating functional genomic variation associated with adaptation and speciation in the wild [1,2]. Consequently, targeting specific genes and genomic regions of interest - rather than random genomic regions - holds a great promise as a shortcut to identify genes involved in phenotypic variation and adaptive divergence [3-5]. Despite a steadily increasing number of completed genome sequences, genomic resources and tools are still very limited for the vast majority of non-model organisms. Therefore, ability to develop molecular markers in or around target genes is essential for application of this approach for non-model organisms. In addition, molecular markers associated with functionally important genes are useful in construction of comparative genetic maps, in which they can be exploited as comparative anchor tagged sequence loci [6,7].

Microsatellites or simple sequence repeats (SSRs) are highly abundant in eukaryotic genomes, accounting for 3-5% of the mammalian genomes [8,9]. Owing to their wide genomic distribution, codominant inheritance and hypervariability, they are widely recognized as one of the most powerful molecular markers in the field of genetics. As a result of the widespread use of SSRs, substantial efforts have been made to devise procedures for developing SSR markers [10,11]. In addition, cross-species transfer of SSR primers is commonly attempted in many taxa [12]. However, SSR markers developed with conventional approaches are derived from the genome more or less in a random manner. Expressed sequence tags (ESTs) are commonly used as an alternative to genomic libraries as a source of SSR markers [11]. SSR markers derived from ESTs have some advantages over those developed from genomic libraries because EST-derived markers can associate with genes of known or putative function, and they exhibit relatively high transferability between closely related species [13-17]. However, SSRs are generally much less abundant in transcribed regions than in non-transcribed regions [18-21] and found typically only in a few percentage of ESTs [15,22-26]. Besides, designing primers requires sufficient flanking sequences, resulting in a considerable reduction in number of ESTs available to develop SSR markers [23,24,26,27]. Therefore, even if a large EST database is available for a target species, ESTs have limitations as a material for development of SSR markers for specific genes.

One way to obtain SSR markers for specific genes and genomic regions in a given species is to use SSR primers developed for the closest relative with a sequenced genome. However, an obvious limitation of this approach is that mutations in SSR flanking sequences will inhibit cross-species amplification success - a problem that is likely to attenuate with an increasing divergence time [28]. In general, success of cross-species transfer is a negative function of the evolutionary distance separating the source and focal species [28-31]. Another crucial issue is related to evolution and persistence of SSRs among different species. Investigations of SSR conservation have demonstrated that several SSRs are retained not only in closely related species, but also in species that have diverged more than 100 million years ago [32-35]. Nevertheless, comprehensive surveys of SSR conservation using aligned sequences of different species have rarely been reported [36,37], making it difficult to estimate the patterns and degree of SSR conservation in different taxa.

For the reasons elaborated above, development of SSR markers for target genes and genomic regions in non-model organisms is challenging. Yet, while the closest relative with a sequenced genome is too distantly related to the focal species, one can take an advantage of the increasing number of completed genomes for different species. For instance, as often used in species for which no direct species-specific sequence information is available, conserved sequences in specific genes and genomic regions of interest can be used to design primer sequences applicable to a wide variety of organisms [e.g. [38]].

Teleosts consist of approximately 28,000 species [39], which correspond to more than half of all living vertebrates. Despite a number of features of evolutionary interest and economical importance, genomic resources and tools are still lacking for most teleost taxa. Currently, genome sequences are available for five species - zebrafish (*Danio rerio*), three-spined stickleback (*Gasterosteus aculeatus*), medaka (*Oryzias latipes*), spotted green pufferfish (*Tetraodon nigroviridis*) and fugu (*Takifugu rubripes*) [40]. The development of genome sequences for three-spined sticklebacks has made great contribution to an understanding of the genetic architecture of several phenotypic traits [41-44]. Because three-spined and nine-spined (*Pungitius pungitius*) sticklebacks exhibit similar ecological and morphological characteristics [45], these species provide an opportunity to study whether the same genes or genomic regions are responsible for phenotypic variation of certain traits and adaptive divergence in different lineages. This would facilitate a molecular understanding of the parallel evolution of these species, which have diverged more than 10 million years ago - equivalent to 5-10 millions of generations [46,47]. A potentially effective strategy to this end would be to develop SSR markers targeting functionally important genes.

The main objective of this study was to develop a large set of SSR markers targeting specific genes and genomic regions for a non-model organism - the nine-spined stickleback - in which genome sequences and ESTs are not yet available. To this end, two strategies were adopted. First, we tested cross-species utility of 158 SSR primer sets for functionally important genes originally developed in three-spined sticklebacks together with 188 SSR markers derived from genomic libraries and ESTs. Secondly, we investigated the potential utility of available sequenced fish genomes to develop SSR markers for functionally important genes in nine-spined sticklebacks. To address prospects for this approach, the patterns and degree of SSR conservation were examined in three-spined and nine-spined sticklebacks using their aligned sequences.

## Results and discussion

### Cross-species utility of three-spined stickleback primers

Out of the 158 SSR markers for functionally important genes (gene-based SSRs), 27 showed robust and specific amplification within the expected size range in nine-spined sticklebacks (Table 1, see also Additional files 1 and 2), resulting in a low level (17.1%) of cross-species amplification. In contrast, amplification success was 34.7% (35 out of 101) and 40.2% (35 out of 87) in the SSR markers derived from genomic libraries (genomic SSRs) and ESTs (EST-derived SSRs), respectively (Table 1). The tendency for higher amplification success with the EST-derived SSRs than with the genomic SSRs is in agreement with the results of previous studies [48-51] - finding which has been explained by high sequence conservation in coding regions [13-15].

**Table 1:** Cross-species amplification of three-spined stickleback SSR primers in nine-spined sticklebacks.

SSR type	Primer site	*N*	Amplified	Polymorphic
Gene-based	All	158	27 (17.1%)	16 (10.1%)
	Exonic pair	9	7 (77.8%)	4 (44.4%)
	Intronic pair	53	2 (3.8%)	1 (1.9%)
	Intergenic pair	55	9 (16.4%)	7 (12.7%)
	Other combinations	41	9 (22.0%)	4 (9.8%)
Genomic	All	101	35 (34.7%)	26 (25.7%)
	Exonic pair	1	1 (100%)	1 (100%)
	Intronic pair	34	14 (41.2%)	10 (29.4%)
	Intergenic pair	58	16 (27.6%)	11 (19.0%)
	Other combinations	6	3 (50.0%)	3 (50.0%)
	Unknown	2	1 (50.0%)	1 (50.0%)
EST-derived	All	87	35 (40.2%)	21 (24.1%)
	Exonic pair	14	7 (50.0%)	6 (42.9%)
	Intronic pair	11	4 (36.4%)	1 (9.1%)
	Intergenic pair	47	17 (36.2%)	10 (21.3%)
	Other combinations	15	7 (46.7%)	4 (26.7%)

Factors affecting cross-species amplification success were assessed using the 388 SSR markers. A hierarchical generalized linear model (GLM) revealed a significant influence of SSR type (cf. gene-based, genomic vs. EST-derived SSRs) on amplification success (F_2,331 _= 8.28, *P *= 0.016). In addition, amplification success was significantly affected by primer site (cf. exonic, intronic, intergenic vs. other combinations; F_3,331 _= 16.43, *P *< 0.001). Across the three SSR types, amplification success was high for the SSR markers in which both forward and reverse primers were located in exonic regions (62.5%, 15 out of 24), whereas it was lower if primers were located either in intronic (20.4%, 20 out of 98) or intergenic regions (26.3%, 42 out of 160; Table 1). This effect was particularly obvious for the gene-based SSRs with intronic primers, in which case the amplification success was very low (3.8%, two out of 53; Table 1). As for EST-derived SSRs, trinucleotide SSRs are the most abundant repeat motif in ESTs and tend to be found in coding regions, whereas dinucleotide SSRs are often found in untranslated regions [48]. The fact that most of the EST-derived SSRs used in our study are dinucleotide repeats (85 out of 87) suggests that a number of the EST-derived SSRs might be located in untranslated regions. While in theory EST-derived SSRs should be located in exonic regions, 54 (out of 87) SSRs were located in intergenic regions according to the Ensembl genebuild. This inconsistency could be due to artifacts such as prediction errors and contamination of cDNA libraries with genomic DNA. Nevertheless, the result that amplification success tended to be higher for the EST-derived SSRs with exonic primers (50.0%, seven out of 14) than for those with intergenic primers (36.2%, 17 out of 47) might, at least in part, result from the fact that sequence homology is less in untranslated regions and increase toward the start codon of the coding regions in related species [51].

While the effects of SSR type and primer site were significant, amplification success was not significantly associated with average primer length (F_1,331 _= 1.09, *P *= 0.297) or average GC content (F_1,331 _= 3.12, *P *= 0.077). Similarly, amplification success was independent of differences in GC content (F_1,331 _= 0.00, *P *= 0.993) and melting temperature (F_1,331 _= 0.53, *P *= 0.465) between primers within a given primer pair. In addition, there was no association between amplification success and expected PCR product size (F_1,331 _= 0.393, *P *= 0.531). While the positive effect of average melting temperature appeared to be significant (F_1,331 _= 5.82, *P *= 0.016), no clear difference of melting temperature was found among the SSR types. Based on these results, it is unlikely that differential amplification success among the three sets of SSR markers stemmed from different primer conditions. In fact, all of the SSR markers were successfully amplified under the same PCR and DNA conditions in three-spined sticklebacks. Multiple gene copies are known to exist in several functionally important genes [52-55]. Rather than primer conditions, divergence of functionally important genes might be the cause of low cross-species amplification success of the gene-based SSRs.

In the gene-based SSRs, polymorphism was found at 16 out of the 27 amplifying loci (59.3%; Table 1). This rate was similar to that observed in the genomic SSRs (74.3%, 26 out of 35) and EST-derived SSRs (60.0%, 21 out of 35; Table 1). In total, 63 out of the 97 amplified loci exhibited polymorphism in Fennoscandian populations (Additional file 1). For the amplified loci, incidence of polymorphism was independent of SSR type (GLM, F_2,90 _= 3.37, *P *= 0.185), SSR location (cf. exonic, intronic vs. intergenic regions; F_2,90 _= 5.11, *P *= 0.078) and SSR repeat motif (cf. di- vs. trinucleotide repeats; F_1,90 _= 0.02, *P *= 0.876). The relatively high proportion of polymorphic loci across the different SSR types and SSR locations suggests that several SSRs are conserved in three-spined and nine-spined sticklebacks. For the polymorphic loci of gene-based SSRs, an average of 8.8 alleles per locus (range = 2-38) were identified in the three populations (Additional file 1). This value was equivalent to that obtained in the genomic SSRs (9.3) and EST-derived SSRs (8.3; Additional file 1). Average heterozygosity varied from 0.19 to 0.55 in the gene-based SSRs, from 0.07 to 0.64 in the genomic SSRs and from 0.14 to 0.63 in the EST-derived SSRs among the three populations (Additional file 1). MICRO-CHECKER analyses did not indicate the presence of null alleles, with the possible exceptions of the CLCN7, GS1, Gac7080P and Stn18 in the Baltic Sea, the Stn127 and GAest41 in the Lake 1 and the GAest16 in the Pyöreälampi. There was no evidence for deviations from Hardy-Weinberg equilibrium at any locus in any of the populations.

In general, our results demonstrate that cross-species utility of SSR primers for functionally important genes is less efficient as compared to that of genomic and EST-derived SSR markers. This is attributed to limited amplification success rather than a low incidence of polymorphism. Therefore, the development of species-specific primers would be necessary for obtaining SSR markers for functionally important genes.

### SSR conservation in sticklebacks and marker development

**Figure 1:**
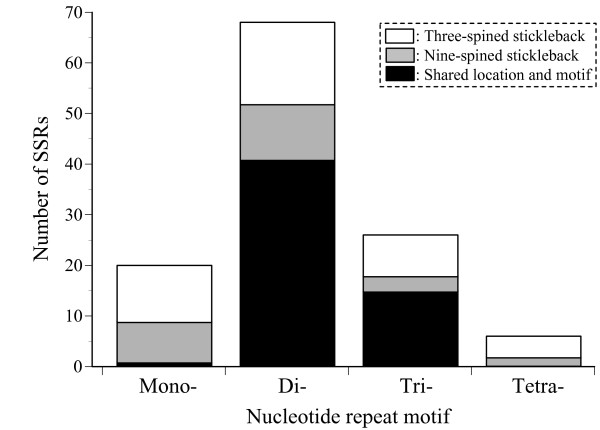
**Number of SSRs observed in 70 genomic regions in three-spined and nine-spined sticklebacks**. Open, unique SSRs for three-spined stickleback; gray, unique SSRs for nine-spined stickleback; black, SSRs of shared motif and location between these species.

To investigate the potential utility of available sequenced fish genomes for SSR marker development in nine-spined sticklebacks, we surveyed SSRs within and around 67 functionally important genes in the three-spined stickleback genome and designed 70 primer sets for amplification and sequencing of these SSR regions using the conserved sequences determined by sequenced fish genomes (Additional file 3). The PCR product size of respective genomic regions obtained in nine-spined sticklebacks was concordant with that estimated from the three-spined stickleback genome (Additional file 4). All of the sequences of nine-spined sticklebacks for the 70 regions exhibited the highest BLAST hit scores in the target regions and high homologies to the sequences of the three-spined stickleback (Additional file 4). Out of the 70 genomic regions representing 44,084 bp, 49 contained at least one SSR in nine-spined sticklebacks (Additional file 4). The total number of SSRs observed in these regions was 81, including 9 mono-, 52 di-, 18 tri- and two tetranucleotide motifs (Figure 1). In the three-spined stickleback genome, 96 SSRs were found in the 70 homologous regions, including 12 mono-, 57 di-, 23 tri- and four tetranucleotide motifs (Figure 1). Out of the 81 SSRs found in nine-spined sticklebacks, 64 were identified at the same locations as those of the three-spined stickleback (Additional file 4). In addition, 57 out of the 64 SSRs exhibited the same motifs as those of the three-spined stickleback (Figure 1, see also Additional file 4), indicating that a large proportion of SSRs are conserved in the genomes of these species. Our results also demonstrated that SSRs with di- and trinucleotide repeat motifs are highly conserved but those with mononucleotide repeat motifs are less so (Figure 1). Hence, the level of SSR conservation may differ among SSRs differing in repeat motif type.

**Figure 2:**
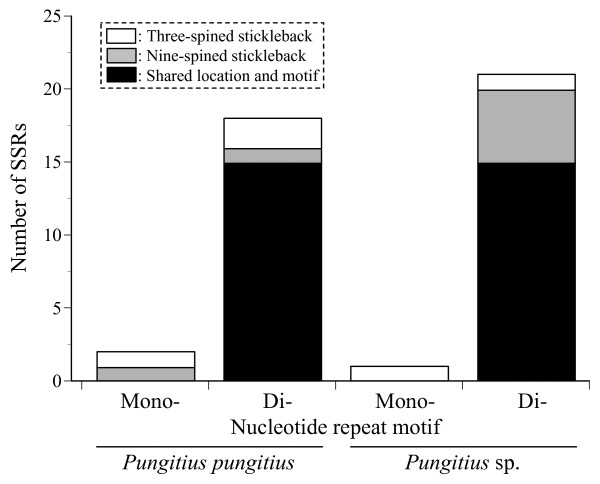
**Number of SSRs observed in 32 genomic regions in three-spined sticklebacks and *Pungitius *species**. Open, unique SSRs for three-spined stickleback; gray, unique SSRs for nine-spined stickleback; black, SSRs of shared motif and location between these species.

To further address SSR conservation in stickleback species, we investigated if SSRs randomly derived from genomic libraries of *Pungitius *species are found at the homologous genomic locations of three-spined sticklebacks. For this analysis, we used publicly available SSR and flanking sequences of *Pungitius pungitiu*s (i.e. nine-spined stickleback) [56] and *Pungitius *sp. [57] - so called the Omono-type, which has been regarded as an independent species from *Pungitius pungitiu*s based on the biological species concept [58]. The 13 *Pungitius pungitius *sequences (5,310 bp) contained one mono- and 16 dinucleotide motif SSRs. In the three-spined stickleback genome, 18 SSRs were identified in the homologous regions, including one mono- and 17 dinucleotide motifs. Out of the 18 SSRs identified in the three-spined stickleback, 15 (83.3%) exhibited the same motifs at the same locations as in *Pungitius pungitius *(Figure 2). In the 19 *Pungitius *sp. sequences (4,117 bp) containing 20 dinucleotide motif SSRs, 17 SSRs were identified in the homologous regions of the three-spined stickleback genome, including one mono- and 16 dinucleotide motifs (Figure 2). Out of the 17 SSRs identified in the three-spined stickleback, 15 (88.2%) exhibited the same motifs at the same locations as in *Pungitius *sp. (Figure 2). The comparative analyses of randomly selected *Pungitius *SSRs in the three-spined stickleback genome further indicated a high degree of SSR conservation in stickleback species.

While several studies have reported conservation of single SSRs between different taxa [32-35,59], a comprehensive survey of SSR conservation is limited to a comparison of human (*Homo sapiens*) and chimpanzee (*Pan troglodytes*) [36,37], which have diverged six million years ago [60]. According to Vowles and Amos [37], 70% of human SSRs are homologues in chimpanzees. Our results demonstrated that a similar proportion (70% for randomly selected motifs) of SSRs are retained in three-spined sticklebacks and *Pungitius *species despite longer divergence time (cf. more than 10 million years) [46] and much shorter generation times (cf. one or two years) [47].

Based on the sequences obtained in nine-spined sticklebacks, species-specific primer sets were designed to amplify 58 SSRs targeting 57 functionally important genes (Table 2). Among them, polymorphism was identified at 41 loci (Table 2) in Fennoscandian populations. On average, 7.7 alleles per locus (range = 2-27) were identified across the three populations (Table 3). Average heterozygosity was 0.57 in the Baltic Sea, 0.37 in the Lake 1 and 0.06 in the Pyöreälampi (Table 3). There was no indication for the presence of null alleles, with the possible exceptions of the Ppgm40 and Ppgm50 in the Baltic Sea and the Ppgm52 and Ppgm56 in the Lake 1. Deviations from Hardy-Weinberg equilibrium were not observed at any locus in any of the populations.

**Table 2:** SSR markers for functionally important genes in nine-spined sticklebacks and their polymorphism (*P*) in three populations.

Gene ID	SSR marker						GenBank accession no
		
	Locus	Repeat motif	Forward primer (5'-3')	Reverse primer (5'-3')	*T*_a _(°C)	*P*	
*ACAPRa*	Ppgm1	(CTC)_12_	AGCTGCCATTTTAAATCCTCCTC	CTCACCATGATGGAAGCCAC	53	No	GU553378
*ACAPRb*	Ppgm2	(AACT)_5_	GGTCTGCCAGGTCATTTCTC	AACGGCACTCATCTGGTTAGT	53	Yes	GU553379
*AE1*	Ppgm3	(GA)_8_GG(GA)_5_	AACACATGACATCACTGCAGC	ACAGGTAAGTCAGTTGTTTCAGG	53	Yes	GU553380
*AQP9*	Ppgm4	(TGT)_6_	AGAAAATAAGCAGCCGTAGC	TGCACGTAAATGGTCTGATT	55	No	GU553381
*ATP1A1*	Ppgm5	(TG)_6_	CCATAGGACGATCACAAG	GAATGAAGTCTTTGTTGTGGGTC	53	No	GU553382
	Ppgm6	(CA)_5_	AGCAGAGCAAAGAACAGGACTC	GATCTCTTTTGCTCTGGAGTTGG	53	No	GU553382
*ATP1A2*	Ppgm7	(TG)_5_/(GT)_12_	TGCATAATGGTCCCCCGTG	AGGCCTTGGCATCCCTG	53	Yes	GU553383
*ATP4A*	Ppgm8	(TC)_5_	TCATTGTAATTTCCGCCTTT	TTTCATCACCAACAGGTAGC	55	Yes	GU553384
*ATP6V1Aa*	Ppgm9	(TG)_6_/(TG)_10_	GACCGATTTCATCTCTGGAC	TGACTCTTTTCCCTCCACTT	55	Yes	GU553385
*ATP6V1Ab*	Ppgm10	(CA)_6_	GCAGGATACCCGCTGTCT	AAGTTCACAAAGGATGCACA	55	Yes	GU553386
*CFTR*	Ppgm11	(CA)_13_	CACTGCTAACACACATCAGC	AAGCGATACCCATCTGTCC	55	Yes	GU553387
*CLCN3*	Ppgm12	(CA)_11_	AGTCGGCATGGGAGTTCAC	GCGATGTCAATCAGGCCG	53	Yes	GU553388
*CLCN4*	Ppgm13	(CTC)_5_	GTTTGAATCCCACAACTTCA	ACTACGTCAACAACCCCAAC	55	No	GU553389
*CLCN7*	Ppgm14	(TG)_10_	CGCTCTGAACAGCTTAAACA	ACGAGAGGGAGTGCATGA	55	Yes	GU553390
*CLCNK*	Ppgm15	(TA)_4_(CA)(TA)	GGCCACCTAGAATTGATGAC	TCAGAGTCCAAACACGAGAA	55	No	GU553391
*CSP2*	Ppgm16	(AAAT)_3_	CGTCGAACTCTACAACCTCC	AGACGTGTTTGTTCATCAGG	55	Yes	GU553392
*DIO1*	Ppgm17	(TC)_9_	CAATCAGGATGTCCAACCA	GCAGCATGGGATGAGAAC	55	Yes	GU553393
*eEF1A1b*	Ppgm18	(AC)_9_	CACTACAGAGTCTAGTCTGAG	TCTTCAGTTAAATGAACCGGTTGC	55	Yes	GU553394
*FERH1*	Ppgm19	(ATC)_5_	TTCTCTCGTTTCTCCAGAGC	GAAATTGACACTGCTGGTTG	55	Yes	GU553395
*FGF6a*	Ppgm20	C_12_	CATCCTTCACCCCAATCTTA	TCTGTCCCCTCTTTCAATGT	55	Yes	GU553396
*FGF18*	Ppgm21	(AG)_12_	TGCCTACTCACACCCACTAA	ATGAGAAATCAATGGAGGGA	55	Yes	GU553397
*GH*	Ppgm22	(GTATA)_3_	TGCGTGGTGTAGTATAGTGTAGTC	AGAGCAACGTCAACTCAACA	55	No	GU553398
*GHRH*	Ppgm23	(AT)_8_	AAGATGAGTTTCCCGCTCTA	TTATTGACTTGACCCTTGACC	55	No	GU553399
*GHR-1*	Ppgm24	(AC)_7_	CCACTACCTCTGCCCTAAAA	TTTCCTTTGGCTTCAATCTC	55	Yes	GU553400
*GHR-2*	Ppgm25	(TA)_6_	TCAACTCTGACCTTCTTGAGG	ACCACAGGTTCACCAAAGAT	55	Yes	GU553401
*GR1*	Ppgm26	A_8_	CTGGTACTGTCCTGATGGAG	TTCTCATAACCACAACTGGC	55	Yes	GU553402
*GR2*	Ppgm27	A_8_	AGAGCACGACAAAACACAGA	AGCAGAAATTGAACAGCACA	55	No	GU553403
*GTF2B*	Ppgm28	(AC)_4_	TGTAATCCCAATACGACGC	AGTATCTGAACCCGCACATT	55	Yes	GU553404
*HPX*	Ppgm29	(AC)_26_	GTGCTTTTAGAAAGACCACCG	TATTACTCTATAGCCGGCAGC	55	Yes	GU553405
*HSP25*	Ppgm30	(AC)_13_	GCAGCGTACATTCTGTTCAAC	GGTTTCTTATGTGGGTGTGAC	53	Yes	GU553406
*HSP47a*	Ppgm31	(CA)_6_	TGCATCATCTGCACTGAAACG	GGGGCAATGATCGTCAATG	55	Yes	GU553407
*HSP70Aa*	Ppgm32	(AC)_15_	CAAAGACCTGCACACACATT	GGGAGCTGTCGATACGTTTA	55	Yes	GU553408
*HSP70Ab*	Ppgm33	(AC)_7_	ATCTACAGGGATACCACAGTAC	TGTTTACTCCGGTCAATGAAACC	53	No	GU553409
*HSP70Ac*	Ppgm34	(TG)_5_	TACTGTTCCACTTGCCCATT	TCACAACTCAGGATCTCGAA	55	No	GU553410
*HSP70B*	Ppgm35	(TC)_14_	TGAGGGTAAAAGCTGTAGCA	ATTATCCCAGAACACTCCCA	55	Yes	GU553411
*HSP90Ab*	Ppgm36	(CA)_5_	GTCAAACCGGACATTAGGAC	CAGACGTGAAACTACGCTTG	55	Yes	GU553412
*HSP90B*	Ppgm37	(TCG)_4_	AGTTATGAAGAAACCGCGTC	GTGATGGCTGTAGCTTGTTG	55	Yes	GU553413
*IGF-I*	Ppgm38	(CGC)_4_	AAGGACGAGCTCGGCTAC	AGGATGCGGCTGCAGATG	53	No	GU553414
*IGF-II*	Ppgm39	(TA)_13_/(TA)_5_	GTTAGGCTTTTACTTGGGTTTCC	TCATTACGCAAGATACAGCTCAG	53	Yes	GU553415
*Kir2.1a*	Ppgm40	(TA)_10_	CTGGAATGACACCAGCCAG	AGTCAGCTCCAAGCTTGTG	53	Yes	GU553416
*Kir2.1c*	Ppgm41	(TG)_7_	CAAAGTGAGCAACAGTGAGC	AAAGGTCGAGGAAGATGATG	55	No	GU553417
*Kir2.2*	Ppgm42	(TC)_12_	ACAGGTACGAAGCGTTTAGC	AGAGCAAAGAAATAGACGGG	55	Yes	GU553418
*MSTNa*	Ppgm43	(CA)_5_	AGTCCGCAATAAGCTCAAAC	ACAAAGCCGTCTAGGTGTTC	55	No	GU553419
*MSTNb*	Ppgm44	(GGA)_8_	GCTGGAGCAGTACGACC	GGTGATGATGGTCTCCGT	55	No	GU553420
*MYHa*	Ppgm45	(TA)_3_C(TA)_3_	AAAGCTCCAAGTAACGCTGT	ACTTTTGTTTCCAATCTGCC	55	No	GU553421
*MYHe*	Ppgm46	(GTAA)_4_	ATGTTAATTGCTTTGTGCGA	TGAAACACAGGAGCTTGAGA	55	Yes	GU553422
*NKCC1b*	Ppgm47	(CA)_13_	ATATGTTCAGCACGCAGCG	GTCAAAGGAGTCTTAGTGAGTG	55	Yes	GU553423
*NPY2Rb*	Ppgm48	(GT)_26_	CACTCAGGAGAAGTGAGGC	GGAACGATTACAGGTACGGAC	53	Yes	GU553424
*NPYP*	Ppgm49	T_10_	CTGCAGCGGACGGGATTAG	AACACAGGACCGACTTTGAGG	53	No	GU553425
*PKMa*	Ppgm50	(GA)_5_	GTGGTACTGCTGGTTGTACT	AAGGTCAAACGGCGTCGC	53	Yes	GU553426
*PVALBb*	Ppgm51	(CA)_6_	AGCTGAACTTTGGTGTGTCTC	GTTGATGTGCATTTATGGGA	55	Yes	GU553427
*SHH*	Ppgm52	G_18_	AGAAACGTGGTTATTGAGGC	CCTGCTCTTTATTGGGTTTT	55	Yes	GU553428
*SLC14*	Ppgm53	(TG)_13_	CCATCATCTCTACACATAATCAAAC	CACTGATTACAGATTGTGTGCTG	53	Yes	GU553429
*SSR1b*	Ppgm54	(TG)_12_	TGTCCTCGCAAAGTTCATAA	TGACCGAGCATTTTACTTGA	55	Yes	GU553430
*T1R3*	Ppgm55	T_18_	ATGCACTGCGTTATCACTCC	GCTTTGTTTAACGTCAGTATTTCG	55	Yes	GU553431
*TAAR*	Ppgm56	(AC)_12_	CAAGGACCACGCTAAAGGTA	GATTTCTTCTTGATGTCCGC	55	Yes	GU553432
*TBX4*	Ppgm57	(GT)_8_	GAGTACGAGCAGGTTTGGTT	GTAACACACACGGTTTTGGA	55	Yes	GU553433
*TTP*	Ppgm58	(AC)_3_(TC)(AC)_3_	GCCACTTATACATGCTCGC	GATTCTCCCTGCCTCACA	55	Yes	GU553434

*T*_a_, annealing temperature.

**Table 3:** Genetic variability of SSR markers for functionally important genes in three populations of nine-spined sticklebacks.

Gene ID	Locus	Total *A*	Baltic Sea				Lake 1				Pyöreälampi			
					
			Size (bp)	*A*	*H*_E_	*F*_IS_	Size (bp)	*A*	*H*_E_	*F*_IS_	Size (bp)	*A*	*H*_E_	*F*_IS_
*ACAPRb*	Ppgm2	3	382-414	3	0.573	-0.017	410	1	0.000	na	414	1	0.000	na
*AE1*	Ppgm3	15	214-260	10	0.779	-0.070	226-308	6	0.560	0.146	228	1	0.000	na
*ATP1A2*	Ppgm7	9	117-143	9	0.774	-0.077	117-139	3	0.576	-0.085	135	1	0.000	na
*ATP4A*	Ppgm8	2	186-196	2	0.042	0.000	186	1	0.000	na	186	1	0.000	na
*ATP6V1Aa*	Ppgm9	7	187-199	7	0.783	-0.065	189-191	2	0.504	-0.324	191	1	0.000	na
*ATP6V1Ab*	Ppgm10	4	102-110	3	0.265	0.058	98-110	2	0.451	-0.293	110	1	0.000	na
*CFTR*	Ppgm11	10	129-159	9	0.593	0.157	139-143	2	0.359	-0.278	149	1	0.000	na
*CLCN3*	Ppgm12	25	185-241	20	0.935	-0.023	194-225	6	0.755	0.118	193-195	2	0.437	-0.241
*CLCN7*	Ppgm14	8	245-266	6	0.626	0.001	254-261	4	0.746	0.107	256	1	0.000	na
*CSP2*	Ppgm16	3	184-192	3	0.490	-0.360	184	1	0.000	na	184	1	0.000	na
*DIO1*	Ppgm17	4	162-166	4	0.545	0.123	162-166	2	0.254	-0.150	166	1	0.000	na
*eEF1A1b*	Ppgm18	5	217-229	5	0.715	0.125	227-229	2	0.223	-0.122	225	1	0.000	na
*FERH1*	Ppgm19	4	217-235	4	0.352	0.054	232-235	2	0.496	-0.007	235	1	0.000	na
*FGF6a*	Ppgm20	14	332-346	12	0.859	-0.063	335-340	4	0.624	0.132	337	1	0.000	na
*FGF18*	Ppgm21	6	331-347	6	0.723	-0.038	335-343	2	0.511	0.021	335	1	0.000	na
*GHR-1*	Ppgm24	5	193-205	3	0.121	-0.030	192-193	2	0.223	-0.122	193-195	2	0.156	-0.070
*GHR-2*	Ppgm25	3	327-333	3	0.492	-0.186	331-333	2	0.082	-0.022	327	1	0.000	na
*GR1*	Ppgm26	3	295-297	3	0.532	0.060	297	1	0.000	na	297	1	0.000	na
*GTF2B*	Ppgm28	2	290	1	0.000	na	288-290	2	0.418	-0.394	290	1	0.000	na
*HPX*	Ppgm29	13	126-169	10	0.746	0.163	137-157	3	0.586	0.289	155-158	2	0.504	0.090
*HSP25*	Ppgm30	4	159-177	4	0.605	0.243	159-175	2	0.478	-0.045	173	1	0.000	na
*HSP47a*	Ppgm31	2	107-109	2	0.042	0.000	107	1	0.000	na	107	1	0.000	na
*HSP70Aa*	Ppgm32	27	283-529	26	0.959	0.094	305-329	4	0.662	0.146	317-319	2	0.466	-0.163
*HSP70B*	Ppgm35	6	324-348	6	0.416	-0.102	324-342	2	0.462	-0.533	324-342	2	0.283	-0.179
*HSP90Ab*	Ppgm36	7	198-232	7	0.629	0.205	204-206	2	0.156	-0.070	204	1	0.000	na
*HSP90B*	Ppgm37	7	244-271	6	0.728	0.085	232-259	3	0.429	-0.068	247	1	0.000	na
*IGF-II*	Ppgm39	8	387-401	8	0.700	0.107	389-397	3	0.160	0.480	393-397	3	0.159	-0.051
*Kir2.1a*	Ppgm40	8	293-327	8	0.713	-0.169	301-321	2	0.511	0.103	297-301	2	0.156	-0.070
*Kir2.2*	Ppgm42	6	289-299	6	0.578	-0.082	291-293	2	0.223	-0.122	289	1	0.000	na
*MYHe*	Ppgm46	6	154-161	5	0.639	0.021	154-159	3	0.508	-0.148	159	1	0.000	na
*NKCC1b*	Ppgm47	10	117-159	10	0.744	-0.065	119-159	3	0.629	0.171	123-125	2	0.042	0.000
*NPY2Rb*	Ppgm48	25	406-471	19	0.918	0.001	423-475	6	0.793	0.002	469	1	0.000	na
*PKMa*	Ppgm50	5	327-334	3	0.518	0.660	327-334	3	0.392	-0.062	327-329	3	0.159	-0.045
*PVALBb*	Ppgm51	5	168-176	5	0.747	0.243	169-176	2	0.042	0.000	169	1	0.000	na
*SHH*	Ppgm52	13	344-356	11	0.856	0.054	353-358	2	0.315	0.603	351-353	2	0.198	-0.100
*SLC14*	Ppgm53	3	230-240	3	0.121	-0.030	236	1	0.000	na	236	1	0.000	na
*SSR1b*	Ppgm54	8	129-145	8	0.544	0.005	135-145	4	0.727	0.083	139	1	0.000	na
*T1R3*	Ppgm55	6	278-285	5	0.558	-0.195	281-286	2	0.082	-0.022	281	1	0.000	na
*TAAR*	Ppgm56	9	123-141	8	0.614	0.050	123-139	4	0.659	0.305	127	1	0.000	na
*TBX4*	Ppgm57	3	278-292	3	0.318	-0.048	278-290	2	0.380	-0.314	290	1	0.000	na
*TTP*	Ppgm58	3	317-321	3	0.291	0.140	317-321	2	0.283	-0.179	317	1	0.000	na

*A*, number of observed alleles; *H*_E_, expected heterozygosity; na, not applied.

### Patterns and degree of SSR variability

The level of SSR variability is known to be associated with repeat motifs due to their different mutation rates [61,62]. In addition, cross-species transfer of SSR primers often results in a lower level of SSR variability in a focal species relative to a source species because of ascertainment bias [63,64]. We investigated the patterns and degree of SSR variability using three Fennoscandian populations. Across the 104 polymorphic loci identified in this study, an average number of alleles per locus and average heterozygosity were 7.4 and 0.60 in the Baltic Sea, 2.6 and 0.32 in the Lake 1, and 1.6 and 0.10 in the Pyöreälampi, respectively. As expected, the levels of SSR variability were significantly dependent on population. The genome-wide survey indicated that genetic variation of the Pyöreälampi is very low, as also shown in a previous study with 11 SSR and one insertion/deletion loci [65]. In our data set, the levels of SSR variability were not dependent on marker origin, SSR type and SSR repeat motif (Table 4). However, a significant influence of SSR location on the levels of allele number and heterozygosity was apparent (Table 4). Across the three populations, average allele number and heterozygosity were 4.7 and 0.41 in exonic regions, 3.3 and 0.30 in intronic regions and 4.2 and 0.36 in intergenic regions, respectively. While the level of SSR variability is known to differ between coding and untranslated regions [66,67], EST-derived SSRs tend to show lower variability than genomic SSRs [50,68]. These differences are thought to arise due to heterogeneous distributions of SSR repeat motifs. However, the higher variability in exonic SSRs than in other SSRs is not explainable by an artifact stemming from different repeat motifs because a majority of the polymorphic SSRs were dimeric repeats independently of their location. Several lines of evidence suggest that SSR variation may affect various traits and be subject to natural selection [21,69,70]. While the potential effect of variable mutation rates can not be ruled out, the heterogeneous distribution of SSR variability observed in this study might be ascribable to natural selection.

**Table 4:** Hierarchical analysis of genetic variability in nine-spined sticklebacks.

Source	Number of alleles					Expected heterozygosity				
		
	Type III SS	df (den)	MS	*F*	*P*	Type III SS	df (den)	MS	*F*	*P*
Marker origin	2.653	1 (298)	2.653	0.222	0.638	0.029	1 (298)	0.029	0.527	0.468
SSR type	16.859	2 (298)	8.430	0.704	0.495	0.079	2 (298)	0.040	0.716	0.489
SSR location	73.246	2 (298)	36.623	3.059	0.048	0.416	2 (298)	0.208	3.782	0.024
SSR motif	36.085	3 (298)	12.028	1.005	0.391	0.073	3 (298)	0.024	0.445	0.721
Population	1978.000	2 (298)	989.000			12.587	2 (298)	6.294		

## Conclusions

Our study demonstrated that a large proportion of SSRs are conserved in the stickleback species which have diverged from a common ancestor more than 10 million years ago [46]. Therefore, the three-spined stickleback genome can be used to predict SSR locations in *Pungitius *species. Our results also suggest that the main limitation of cross-species utility of SSR markers lies in the failure of amplification success probably due to mutations in SSR flanking sequences. While it is possible to predict to some degree the likelihood of amplification success based on the information of primer binding sites, cross-species transferability of SSR primers for functionally important genes is particularly low as compared to that of genomic and EST-derived SSR primers. Yet, SSR markers can be developed for functionally important genes and target genomic regions using the approach outlined in this paper. This approach should be applicable also to other non-model organisms. The SSR markers developed for functionally important genes should be useful to identify genes responsible for phenotypic variation and adaptive divergence in nine-spined sticklebacks, as well as for constructing comparative gene maps of nine-spined and three-spined sticklebacks.

## Methods

### Fish samples

Nine-spined sticklebacks collected from the Baltic Sea (coastal; 60°12' N, 25°11' E), the 'Lake 1' (lake; 67°54' N, 20°50' E) and the Pyöreälampi (pond; 66°16' N, 29°26' E) were used in this study. The fish were sampled with seine nets or minnow traps in 2002 (Lake 1) and 2008 (Baltic Sea and Pyöreälampi). Total DNA was extracted from fin clips stored in 70-99% ethanol with a phenol-chloroform method [71] following proteinase K digestion.

### Cross-species transfer of three-spined stickleback SSR primers

Cross-species utility of three-spined stickleback SSR primers was tested for 158 SSR markers for physiologically important genes (gene-based SSRs) [Y. Shimada, T. Shikano and J. Merilä, unpublished] coupled with 101 markers derived from genomic libraries (genomic SSRs) and 87 markers derived from ESTs (EST-derived SSRs; Additional files 1 and 2) [72-76]. The genomic and EST-derived SSRs were classified according to the source information deposited in GenBank [77]. The following factors potentially affecting cross-species amplification success were scored for each of the makers: SSR marker type (cf. gene-based, genomic and EST-derived SSRs), primer binding site (cf. exonic, intronic, intergenic and other combinations), average primer length, average and difference of GC content and melting temperature in forward and reverse primer pairs, as well as expected PCR product size. Primer binding sites were categorized into exonic, intronic and intergenic regions based on the Ensembl genebuild in the three-spined stickleback genome [40]. Since information on untranslated regions was not available for a number of genes, we did not distinguish between coding and untranslated regions in the analyses. The primer parameters were calculated using BioEdit [78] under the actual PCR conditions (see below). The expected PCR product sizes were calculated based on the three-spined stickleback genome. The role of these factors was evaluated using generalized linear models as implemented in JMP 5 (SAS Inst. Inc.). In these tests, amplification success was treated as a binary dependent variable (successful amplification = 1, failed amplification = 0), SSR type and primer site as factors, and other parameters as covariates. Logit link function was used. For the successfully amplified loci, factors affecting incidence of polymorphism were evaluated using generalized linear models treating SSR type, SSR location (cf. exonic, intronic and intergenic regions) and SSR repeat motif (cf. di- and trinucleotide repeats) as factors. SSR location was categorized into exonic, intronic and intergenic regions based on the Ensembl genebuild in the three-spined stickleback genome. In this test, polymorphic locus was treated as a binary dependent variable (polymorphic = 1, monomorphic = 0) using logit link function.

### SSR primer development in nine-spined sticklebacks

Based on the literature on gene functions in teleosts, we selected 67 genes responsible for significant physiological - such as osmoregulation, thermal response, growth, disease and taste [Y. Shimada, T. Shikano and J. Merilä, unpublished] - and developmental functions [e.g. [41]] (Additional file 3). Genomic locations of these genes were identified in the three-spined stickleback genome following Shimada *et al*. [Y. Shimada, T. Shikano and J. Merilä, unpublished]. In brief, we searched the three-spined stickleback ESTs which correspond to target genes of this species or other teleosts in the GenBank database [77] and mapped them in the three-spined stickleback genome. The genomic range of respective genes was determined according to the Ensembl transcript and Genscan predictions (Additional file 3). Since the genomic region of the *PITX1 *was not available due to partially incomplete sequences of the three-spined stickleback genome, the sequence of this gene (GenBank: AY517634.1) was used.

SSRs were searched in the target genes and their flanking regions in the three-spined stickleback genome using Tandem repeats finder [79]. In order to survey conserved regions for designing amplification and sequencing primers in nine-spined sticklebacks, the sequences of these genomic regions were subject to BLASTN searches against the currently available genome sequences of other teleosts, i.e. medaka, fugu, spotted green pufferfish and/or zebrafish [40]. Conserved regions were determined by aligning the sequences of three-spined sticklebacks and those of other fish species detected by the BLASTN searches. Based on the location of SSRs and conserved regions, primer sequences for nine-spined sticklebacks were designed manually in one genomic region for each target gene, except for the *GHRI *and *IGF-I*, for which two and three regions were used, respectively (Additional file 3).

Two individuals of the Pyöreälampi were used for amplifying and sequencing the target genomic regions. One three-spined stickleback individual from the Baltic Sea (60°12' N, 25°11' E) was used as a positive control. Using a primer pair for respective target regions (Additional file 3), PCR amplifications were carried out in a 20 μl reaction volume consisting of 1× PCR buffer (Bioline), 1.5 mM MgCl_2_, 0.25 mM dNTP (Finnzymes), 0.15 U BIOTAQ DNA polymerase (Bioline), 5 pmol of each primer and approx. 40 ng of genomic DNA. The reactions were performed as follows: an initial degeneration step at 95°C for 3 min, followed by 30 s at 95°C, 30 s at 53-60°C and 60-120 s at 72°C for 35 cycles with a final extension at 72°C for 5 min (see Additional file 3 for optimal PCR conditions in each primer pair). Approximate size of the PCR amplicons was determined by electrophoresis on 1.5% agarose gel with a DNA ladder (GeneRuler™ DNA Ladder Mix, Fermentas). PCR products were purified using exonuclease I (New England Biolabs) and shrimp alkaline phosphatase (Roche) and directly sequenced in both forward and reverse directions with the same primers as those used in the PCRs. The sequencing reactions were performed using the BigDye Terminator v3.1 Cycle Sequencing Kit (Applied Biosystems) according to manufacture's instructions. Cycle sequencing products were purified by ethanol precipitation and analyzed on an ABI 3730*xl *DNA Analyzer (Applied Biosystems).

The sequences in forward and reverse directions of two individuals were aligned using CLUSTAL W [80] as implemented in MEGA 4 [81] and edited by hand. For large PCR amplicons (≥1200 bp), the sequences in forward and reverse directions were separately aligned using two individuals (Additional file 4). As sequences were available only in one direction for four genomic regions even after retrials, sequences for these regions were aligned using two individuals (Additional file 4). The sequences were subject to BLASTN searches against the three-spined stickleback genome to ensure that they are mapped back to the correct locations in the genome. The homologous sequences in three-spined and nine-spined sticklebacks were aligned to compare SSR locations and motifs between them. This comparison was performed using SSRs with minimum repeat numbers of ten, five and four for one (mono-), two (di-) and three or longer (tri-, tetra-, penta- and hexanucleotide) repeat motifs, respectively. To further address SSR conservation in stickleback species, we also investigated if SSRs randomly derived from genomic libraries of *Pungitius *species are found at the homologous locations in the three-spined stickleback genome using publicly available SSR and flanking sequences of *Pungitius pungitiu*s (i.e. nine-spined stickleback) [[56]; GenBank: AB473819-AB473831] and *Pungitius *sp. (Omono type) [[57]; GenBank: AB300827-AB300851]. Out of the 38 sequences, six (GenBank: AB300830, AB300831, AB300841, AB300842, AB300844, AB300849) were excluded from the analyses because of low BLAST hit scores and alignment problems. To develop SSR markers for functionally important genes, primer sets were designed based on the sequences of nine-spined sticklebacks using WebSat [82]. Primer sequences were deposited in GenBank under accession numbers GU553378-GU553434.

### SSR amplification and genotyping

For the cross-species amplification test of three-spined stickleback primers, amplification success and polymorphism were determined using the following three-step procedure. Firstly, amplification was tested using four individuals from the Pyöreälampi and Baltic Sea (two individuals per population) with fluorescent labelled forward primers (FAM, HEX or TET) and GTTT-tailed reverse primers [83]. As a positive control, one individual of the three-spined stickleback was used. PCRs were performed under optimal conditions for three-spined sticklebacks and conducted in a 10 μl reaction volume consisting of 1× PCR buffer (Bioline), 1.5 mM MgCl_2_, 0.2 mM dNTP (Finnzymes), 0.18 U BIOTAQ DNA polymerase (Bioline), 5 pmol of each primer and approx. 20 ng of template DNA. The reactions were performed as follows: an initial degeneration step at 95°C for 3 min, followed by 30 s at 95°C, 30 s at 53°C and 30 s at 72°C for 30 cycles with a final extension at 72°C for 5 min. Amplification success was determined by electrophoresis on 1.6% agarose gel. Secondly, for the loci that showed robust and specific amplification within the expected size range, polymorphism was investigated by genotyping 24 individuals from the Baltic Sea and the Lake 1 (12 individuals per population). For efficient screening, PCRs were carried out using the Qiagen Multiplex PCR Kit (Qiagen) in 10 μl reaction volumes containing 1× Qiagen Multiplex PCR Master Mix, 0.5× Q-Solution, 2 pmol of each primer and approx. 20 ng of template DNA. The reactions were performed by the following cycle: an initial activation step at 95°C for 15 min, followed by 30 s at 94°C, 90 s at 53°C and 60 s at 72°C for 30 cycles with a final extension at 60°C for 5 min. PCR products were visualized with a MegaBACE 1000 automated sequencer (Amersham Biosciences) and their sizes were determined with ET-ROX 550 size standard (Amersham Biosciences). Thirdly, genetic variability of the polymorphic loci identified with the 24 individuals was evaluated by genotyping a total of 24 individuals from each of the Baltic Sea, Lake 1 and Pyöreälampi populations using multiplex PCRs. For the SSR primers developed in nine-spined sticklebacks, polymorphism and genetic variability were evaluated following the procedures for the second and third steps. Since some of the SSR markers yielded clearer allele profiles at an annealing temperature of 55°C, this temperature was used for these loci instead of 53°C (see Table 2 for an optimal annealing temperature for each primer pair). Alleles were scored using Fragment Profiler 1.2 (Amersham Biosciences) with visual inspection and manual corrections of alleles.

### SSR data analyses

Locus and population specific gene diversities (*H*_E_) [84] were estimated using FSTAT 2.9.3 [85,86]. Within population and locus specific *F*_IS _were estimated for each population to detect possible deviations from Hardy-Weinberg equilibrium with 10 000 permutations using FSTAT 2.9.3. Sequential Bonferroni corrections [87] were applied to minimize type I errors. The presence of null alleles was tested using MICRO-CHECKER [88].

Factors affecting the levels of genetic variation were evaluated with general linear models where allele number or heterozygosity was treated as a dependent variable, SSR marker origin (cf. three-spined and nine-spined sticklebacks), SSR type, SSR location and SSR repeat motif (cf. mono-, di-, tri- and tetranucleotide repeats) as fixed factors and population as a random factor. These analyses were performed with JMP 5.

## Authors' contributions

TS conceived the study, contributed to the gene selection and localization, prepared the molecular data, conducted the analyses and wrote the manuscript. JR selected the genes and conducted the molecular work. YS made contributions to the gene selection and localization. JM advised on the statistical analyses and contributed to writing the manuscript. All authors read and approved the final manuscript.

## Supplementary Material

Additional file 1 Genetic variability of three-spined stickleback SSR markers in three populations of nine-spined sticklebacks. Click here for file

Additional file 2 List of three-spined stickleback SSR markers that were not polymorphic or successfully amplified in nine-spined sticklebacks. Click here for file

Additional file 3 Target genes and their function and location in the theree-spined stickleback genome, and PCR and sequencing primers for nine-spined sticklebacks. Click here for file

Additional file 4 Homology of nine-spined stickleback sequences in the three-spined stickleback genome and comparative genomic location of SSRs in these species. Click here for file
