# Supplementary material for: Utility of sequenced genomes for microsatellite marker development in non-model organisms: a case study of functionally important genes in nine-spined sticklebacks (Pungitius pungitius)
Source: BMC Genomics. 2010 May 27;11:334. doi: 10.1186/1471-2164-11-334 (PMC2891615; doi:10.1186/1471-2164-11-334)
Supplement: Additional file 1 — Genetic variability of three-spined stickleback SSR markers in three populations of nine-spined sticklebacks. [file 1471-2164-11-334-S1.PDF]

Additional file 1: Genetic variability of three-spined stickleback SSR markers in three populations of nine-spined sticklebacks

SSR type	Total A	Baltic Sea				Lake 1				Pyöreälampi			
Locus		Size (bp)	A	H_E	F_{IS}	Size (bp)	A	H_E	F_{IS}	Size (bp)	A	H_E	F_{IS}
Gene-based													
ATP1A1	15	179-239	14	0.849	0.027	179-185	2	0.509	0.181	189-197	3	0.508	0.098
CLCN7	15	131-183	14	0.899	0.166	141-153	4	0.703	0.111	151-159	3	0.293	-0.136
FGF8	3	289-295	3	0.086	-0.011	291-295	2	0.511	0.021	291	1	0.000	na
FGF18	3	218-222	3	0.491	-0.273	218	1	0.000	na	218	1	0.000	na
GR1	3	170-172	3	0.553	0.136	171-172	2	0.513	0.187	170-171	2	0.231	-0.128
GS1	17	327-447	14	0.923	0.508	337-393	4	0.716	-0.279	375-393	2	0.223	-0.122
HSP70Ad	2	336-337	2	0.508	0.058	336-337	2	0.190	-0.095	336	1	0.000	na
IGF-II	8	210-224	8	0.700	0.130	212-220	3	0.160	0.480	216-220	3	0.165	-0.054
KCNJ4b	8	102-130	7	0.630	0.272	110	1	0.000	na	110-136	2	0.339	0.262
MYHb	2	365-366	2	0.043	0.000	365-366	2	0.481	-0.188	366	1	0.000	na
NHE2b	2	273-275	2	0.382	-0.090	273	1	0.000	na	275	1	0.000	na
NHE2c	16	175-213	13	0.902	-0.012	191-201	4	0.649	-0.072	191-195	2	0.042	0.000
NHE3	2	309	1	0.000	na	309-312	2	0.082	-0.022	309	1	0.000	na
NPY2Rb	38	214-316	23	0.880	-0.042	252-324	9	0.817	0.009	254-328	16	0.824	0.007
PVALBa	4	217-225	4	0.452	-0.106	219-225	2	0.283	-0.179	219-225	2	0.190	-0.095
UDPGT	2	251-259	2	0.480	0.132	251-259	2	0.176	-0.081	251-259	2	0.162	-0.073
Genomic													
Gac1097P	3	113-117	3	0.584	0.216	113	1	0.000	na	113-117	2	0.400	-0.353
Gac1125P	6	132-148	6	0.749	-0.001	140-146	3	0.508	-0.148	140	1	0.000	na
Gac4174P	30	207-323	26	0.966	-0.035	241-397	7	0.770	-0.028	229	1	0.000	na
Gac7080P	18	180-218	17	0.918	0.147	182-198	5	0.331	0.118	182	1	0.000	na
Stn18	10	112-149	10	0.738	0.261	112	1	0.000	na	112	1	0.000	na
Stn19	4	158-176	4	0.612	-0.157	158-176	2	0.462	-0.533	158-176	2	0.292	-0.189
Stn46	2	241-243	2	0.509	-0.064	241	1	0.000	na	241	1	0.000	na
Stn49	4	161-173	4	0.266	-0.095	169	1	0.000	na	169	1	0.000	na
Stn52	17	165-201	15	0.897	0.117	171-175	3	0.506	0.177	177-183	2	0.042	0.000
Stn79	7	133-213	7	0.274	0.238	133	1	0.000	na	133	1	0.000	na
Stn96	8	207-241	8	0.687	-0.091	235-241	2	0.509	-0.064	235	1	0.000	na
Stn100	5	176-184	5	0.614	-0.018	176-180	2	0.361	-0.040	176	1	0.000	na
Stn102	5	137-149	5	0.199	0.164	145	1	0.000	na	145	1	0.000	na
Stn108	4	103-117	4	0.616	-0.082	105-111	2	0.487	-0.112	111	1	0.000	na
Stn127	25	232-338	14	0.909	-0.009	296-346	12	0.839	0.223	248-250	2	0.042	0.000
Stn148	3	195-203	3	0.521	0.040	195-197	2	0.082	-0.022	195	1	0.000	na
Stn156	3	143-151	3	0.353	0.292	145	1	0.000	na	151	1	0.000	na
Stn173	6	114-124	6	0.661	-0.008	116-120	2	0.284	0.121	116	1	0.000	na
Stn194	17	93-141	16	0.864	-0.108	95-119	4	0.601	-0.041	95-119	3	0.520	-0.042
Stn195	5	155-171	5	0.578	0.207	155-171	3	0.122	0.319	171	1	0.000	na
Stn198	5	207-212	5	0.716	0.011	207-210	2	0.120	-0.045	207	1	0.000	na
Stn222	21	138-196	20	0.940	-0.019	146-156	3	0.543	-0.303	148-150	2	0.324	0.329
Stn223	8	195-219	8	0.798	0.112	211-219	3	0.082	-0.011	211-215	2	0.042	0.000
Stn253	14	104-140	11	0.846	-0.034	114-136	4	0.697	-0.196	132-136	2	0.042	0.000
Stn315	9	107-145	9	0.662	0.081	129	1	0.000	na	129	1	0.000	na
Stn328	2	149-151	2	0.225	0.258	151	1	0.000	na	149	1	0.000	na
EST-derived													
Stn71	3	151-159	3	0.195	0.144	151	1	0.000	na	151	1	0.000	na
Stn89	3	168-174	3	0.570	-0.097	172-174	2	0.514	0.352	174	1	0.000	na
Stn124	6	225-237	5	0.608	-0.165	225-231	2	0.322	0.055	233	1	0.000	na
Stn130	6	194-204	6	0.622	-0.071	196	1	0.000	na	196	1	0.000	na
Stn163	6	136-146	6	0.641	-0.105	138	1	0.000	na	138	1	0.000	na
Stn196	4	192-200	4	0.530	0.056	192-196	3	0.159	-0.045	196	1	0.000	na
Stn257	16	225-267	16	0.875	0.056	227-249	3	0.159	-0.051	227	1	0.000	na
GAest3	5	154-166	5	0.767	0.077	154-160	2	0.312	0.064	158-160	2	0.121	0.657
GAest6	7	244-270	7	0.491	0.151	244-256	2	0.337	0.011	244	1	0.000	na
GAest7	5	287-300	5	0.422	-0.086	287	1	0.000	na	287-299	3	0.610	0.248
GAest14	9	117-143	9	0.821	-0.015	129	1	0.000	na	129	1	0.000	na
GAest16	27	165-243	19	0.918	0.093	195-269	8	0.688	-0.029	221-269	8	0.773	0.269
GAest30	11	232-262	11	0.846	0.064	232-238	3	0.430	-0.356	232	1	0.000	na
GAest34	16	195-225	15	0.917	0.091	197-217	5	0.439	-0.043	213-219	3	0.262	0.363
GAest35	8	235-259	8	0.821	0.035	247	1	0.000	na	247	1	0.000	na
GAest41	9	289-305	7	0.734	-0.022	295-303	3	0.302	0.449	289-291	2	0.042	0.000
GAest50	3	80-86	3	0.082	-0.011	86	1	0.000	na	86	1	0.000	na
GAest51	14	196-216	13	0.883	0.022	198-210	3	0.636	-0.114	198-227	2	0.491	0.236
GAest66	5	186-192	4	0.727	-0.031	184-192	3	0.452	0.078	192	1	0.000	na
GAest67	9	174-200	7	0.520	0.038	184-188	2	0.438	-0.045	184-198	3	0.620	-0.143
GAest82	3	111-127	3	0.307	-0.132	111-123	2	0.156	-0.070	123	1	0.000	na

A , number of observed alleles; H_E, expected heterozygosity; na, not applied.
