# Supplementary material for: Utility of sequenced genomes for microsatellite marker development in non-model organisms: a case study of functionally important genes in nine-spined sticklebacks (Pungitius pungitius)
Source: BMC Genomics. 2010 May 27;11:334. doi: 10.1186/1471-2164-11-334 (PMC2891615; doi:10.1186/1471-2164-11-334)
Supplement: Additional file 2 — List of three-spined stickleback SSR markers that were not polymorphic or successfully amplified in nine-spined sticklebacks. [file 1471-2164-11-334-S2.PDF]

Additional file 2: List of three-spined stickleback SSR markers that were not polymorphic or successfully amplified in nine-spined sticklebacks

SSR type	Amplification	*N*	Locus										
Gene-based	Monomorphic	11	AQP9	ATP4A	CLCNK	DCT	HSP70Ab	KCNA5a	PER1	PRL-R	RhBG	SLC4A10a	HPX
	Failed	131	ACAPRa	ACAPRb	AhR1Aa	AhR1Ab	AhR2Aa	AhR2Ab	AQP3	AQP4	ATP1A2	ATP1A3a	ATP1A3b
			ATP2C1	ATP6V1Aa	ATP6V1Ab	CASP6	CASR	CFTR	CLCN3	CLCN4	CLCN5	CLOCKa	CLOCKb
			CSP1	CSP2	CSP3	EEF1A1a	EEF1A1b	ESR1	eUT-C	FABP2	FERH1	FERH2	FGF2
			GH	GHRH	GHR-I	GHR-II	GLUR1	GR2	GTF2B	HIF1Aa	HIF1Ab	HSC70	HSF2
			HSP25	HSP27	HSP30	HSP47b	HSP60	HSP70Aa	HSP70Ac	HSP90Ab	HSP90B	IGF-I	IGFBP1
			IL8	IL12B	IRS2	KCNA3	KCNA5b	KCNC3	KCNJ4a	KCNJ6	KCNJ9	KCNJ10	KCNJ12
			KCNJ15	Kir2.1a	Kir2.1b	Kir2.2	LDH-AS	LEP	LEPR	LPP2	MET	MHC-IIB	MKP1a
			MKP1b	MKP8	MSTNa	MSTNb	MYHc	MYHd	MYHe	MYOD	MYOG	NBC1a	NBC1b
			NCC	NHE2e	NHE2f	NKCC1a	NKCC1b	NPY2Ra	NPYP	PKMa	PKMb	PVALBb	RASGRP1
			RhCG1	RhCG2a	RHOGTP8	RIPK2	RPEST	SCI	SGK1	SHH	SLC4A7a	SLC4A7b	SLC4A10b
			SLC14	SLP	SOCS3	SOD1	SPG1	SSR1a	SSR1c	SSR5	T1R2-1	T1R2-3	T1R3
			TAAR	TCTP	TRAa	TRAb	TRB	TYR1	TYR2	VEGF2a	VEGF2b	VEGFp	
Genomic	Monomorphic	9	Gac7033P	Stn21	Stn32	Stn123	Stn167	Stn177	Stn185	Stn299	Stn302		
	Failed	66	Gac4115P	Gac4160P	Gac7148P	Stn1	Stn3	Stn8	Stn9	Stn12	Stn23	Stn29	Stn37
			Stn38	Stn42	Stn57	Stn59	Stn61	Stn64	Stn67	Stn70	Stn73	Stn82	Stn83
			Stn84	Stn88	Stn90	Stn110	Stn118	Stn125	Stn132	Stn149	Stn153	Stn154	Stn157
			Stn159	Stn168	Stn179	Stn187	Stn190	Stn191	Stn199	Stn200	Stn201	Stn208	Stn214
			Stn216	Stn247	Stn248	Stn252	Stn254	Stn261	Stn268	Stn280	Stn283	Stn289	Stn290
			Stn291	Stn301	Stn319	Stn321	Stn322	Stn324	Stn325	Stn327	Stn334	Stn387	Stn389
EST-derived	Monomorphic	14	Stn5	Stn20	Stn34	Stn74	Stn119	Stn122	Stn164	Stn235	Stn240	GAest11	GAest47
			GAest63	GAest84	GAest87								
	Failed	52	Stn15	Stn17	Stn22	Stn26	Stn30	Stn50	Stn51	Stn56	Stn65	Stn76	Stn78
			Stn86	Stn93	Stn99	Stn103	Stn134	Stn135	Stn138	Stn146	Stn158	Stn160	Stn170
			Stn174	Stn178	Stn204	Stn205	Stn219	Stn263	Stn279	Stn306	Stn308	GAest4	GAest8
			GAest15	GAest17	GAest19	GAest21	GAest26	GAest29	GAest31	GAest32	GAest36	GAest42	GAest43
			GAest49	GAest55	GAest56	GAest57	GAest61	GAest71	GAest73	GAest80			
