# Supplementary material for: Utility of sequenced genomes for microsatellite marker development in non-model organisms: a case study of functionally important genes in nine-spined sticklebacks (Pungitius pungitius)
Source: BMC Genomics. 2010 May 27;11:334. doi: 10.1186/1471-2164-11-334 (PMC2891615; doi:10.1186/1471-2164-11-334)
Supplement: Additional file 3 — Target genes and their function and location in the theree-spined stickleback genome, and PCR and sequencing primers for nine-spined sticklebacks. [file 1471-2164-11-334-S3.PDF]

Additional file 3: Target genes and their function and location in the thereе-spined stickleback genome, and PCR and sequencing primers for nine-spined sticklebacks

Target genes and their positions in three-spined stickleback genome					Genomic regions and PCR conditions for sequencing					
Gene ID	Gene name	Function	LG	Position (bp)	Sequencing region	Forward primer (5'–3')	Reverse primer (5'–3')	T_a (°C)	Ext. time	Exp. size (bp)
ACAPRa	Pituitary adenylate cyclase activating polypeptide receptor subtype 1	GR	III	13,117,473-13,151,610	Within gene	ACGGAACTCCCACCATCTG	GGATTCGTTGCCTCCGATGTC	53	1'30	863
ACAPRb	Pituitary adenylate cyclase activating polypeptide receptor subtype 2	GR	VIII	4,746,170-4,754,936	Within gene	GACACAAAGAGGTTCATGTGGATGAAG	TGCACTGAGTTCGGCTGG	60	2'00	744
AE1	Band 3 anion exchanger 1	OR	XI	7,093,673-7,107,832	9kbp from gene	GCTCAGCGCTGCTCAGTTC	GAGGAGAAGCAGAGGCAGTAC	60	1'30	820
AQP9	Aquaporin 9	OR	II	11,320,539-11,328,792	Within gene	CGCAGTTCAGACCCATGGACAC	GGGCTTTTGCTGGATCCTGTGC	60	1'30	1434
ATP1A1	Na^+/K^+-ATPase alpha-subunit isoform 1	OR	I	21,699,651-21,730,178	Within gene	TCCATAGGACGATCACAAGTT	ACCTGTTTACAGAACTTGACCCATTC	58	1'30	1471
ATP1A2	Na^+/K^+-ATPase alpha-subunit isoform 2	OR	III	15,239,341-15,268,451	Within gene	CTGAGCCAAGTCAACCCCAGG	TCTCAGTGTGGTTCCTCAGCAGG	60	1'30	523
ATP4A	H^+/K^+-ATPase alpha subunit	OR	XX	10,304,814-10,312,372	Within gene	CCTTCAACTCCACCAACAAGT	GTGCCCCTTTCATCACCA	60	1'00	324
ATP6V1Aa	V-type H^+-ATPase subunit A subtype a	OR	XXI	7,736,424-7,747,045	Within gene	GGAGAAATCCTCTACAGGATCAGCTC	AGTACATCTGACTCTTTTCCCTCCAC	60	1'30	535
ATP6V1Ab	V-type H^+-ATPase subunit A subtype b	OR	VII	22,247,011-22,257,290	Within gene	GTAAGCAGGATACCCGCT	TGAGCATGATGGCCGACT	60	1'30	195
CASR	Extracellular calcium sensing receptor	OR	VII	22,294,868-22,319,211	2kbp from gene	ACCCCTGGAAGCAGTGGAT	GGCGAAAGTTGGACACCTC	60	1'30	1648
CFTR	Cystic fibrosis transmembrane conductance regulator	OR	XIX	10,185,959-10,207,752	Within gene	TCCGACAGGATGACGGTGCAG	GGAGGCAACGTGCTGAGCAAC	60	1'00	827
CLCN3	CLC chloride channel isoform 3	OR	VII	2,422,683-2,438,993	Within gene	ACCTGTTGGACTTCCTGGAGGAG	AAGGTGGTCTCGTTGGACGTCC	56	2'00	998
CLCN4	CLC chloride channel isoform 4	OR	I	27,636,316-27,641,146	Within gene	GCCGGCACCAGTTCTTGAAGATGA	ATCTCGGAGCTCTTCAACGACTGC	56	1'30	441
CLCN7	CLC chloride channel isoform 7	OR	XI	14,735,658-14,744,755	Within gene	CCCAGGTGATATCTGGCCAAGTC	CCGATCCAGAGCATCCACGTCT	60	2'00	513
CLCNK	Kidney-specific chloride channel	OR	XII	5,800,399-5,809,051	Within gene	AAATCTCTGCAGGAAGGGTCT	AGAGCTCCACTGTCTGC	60	1'30	471
CSP1	Cold shock protein 70 kDa subtype 1	TR	XI	4,631,751-4,642,238	Within gene	CCTTGTTGGCCAGCTTCTTGTTGAG	GTGCACTGTAGCTTTAAGTTTTGCGAC	58	1'30	414
CSP2	Cold shock protein 70 kDa subtype 2	TR	VII	4,339,418-4,342,520	Within gene	GTTTGTTGGGCGCGTAGCG	CAAAGAAGACGTGTTTGTTCATCAGG	58	1'30	371
DIO1	Type I iodothyronine deiodinase	MR	VIII	15,377,962-15,379,810	6kbp from gene	TTCATGTCGCCTCGCTTTGC	ATGTTGGGCAGCATGGGATG	58	1'30	200
eEF1A1b	Eukaryotic elongation factor 1 alpha isoform 1 subtype 2	GR	IX	9,892,172-9,902,175	5kbp from gene	GAGGAAAAGGTTTTGGCTCGGCTG	CATCAAGAGCCTGCAGACCAGGT	58	1'30	350
FERH1	Ferritin H subunit 1	TR	II	8,646,351-8,651,500	4kbp from gene	AGAAACGACTCCATCTCGGACA	ATGAAGAGCAGATCTGGCAGGAG	58	1'30	433
FGF2	Fibroblast growth factor isoform 2	GR	IV	3,331,137-3,335,024	1kbp from gene	GGGAACTTCCACGCATTCTTTGTCTG	GCAGGTGCAGTTGTTGATGAATC	58	1'30	1552
FGF6a	Fibroblast growth factor isoform 6 subtype 1	GR	XIX	19,484,670-19,489,133	Within gene	TCACCACTCCTCTGTCCACCGT	TCTACTGCAACGTGGGCATCGG	53	1'30	691
FGF18	Fibroblast growth factor 18	GR	IV	10,277,803-10,300,572	Within gene	AGGTGCGAGACACCATGAGTCG	CGTGACCTTAGTCAACAAAGGGGTC	58	2'00	987
GH	Growth hormone	GR	XI	16,056,253-16,072,008	Within gene	CGACTGGGAAACTCCCAGGA	CCACCTGCTCGCTCAGAGACT	60	2'00	1229
GHRH	Growth hormone releasing hormone	GR	XXI	9,519,809-9,554,290	Within gene	AGTGGCCCTACAATGCTG	ATTGGCTCCAAAGATTAGACG	53	1'30	452
GHR-1	Growth hormone receptor 1	GR	XIII	5,674,354-5,688,758	2kbp from gene	GAATATATTAAGACATTTCTGCAACAGC	GAACAAGTAGAACGACTGTGAAAG	54	1'00	1226
					3kbp from gene	AGAGGGAGGCTCGGCTCAG	GTGCAGAGCGATGATGAATGTGG	58	1'30	489
GHR-2	Growth hormone receptor 2	GR	XIV	10,388,550-10,398,180	Within gene	TGCCTCCTGTTCCTGGACCT	AACTCCACCCAGGGGTCG	60	2'00	883
GR1	Glucocorticoid receptor isoform 1	OR	IX	11,863,234-11,888,356	Within gene	TGCGGGAGTATTTGTTGCCACGG	CATGGACCGAGCCCAGATGG	60	2'00	784
GR2	Glucocorticoid receptor isoform 2	OR	VII	23,164,545-23,201,619	Within gene	TGAAGGATGGAGAGCACGA	GGTGGAACATTCCAAGCAGAA	56	1'30	302
GTF2B	General transcription factor IIB	OR	III	8,925,781-8,935,595	3kbp from gene	CCTTCTTTGCAAAGCCCAAAGTC	TTCCTGGTCAACCTGTTTGCGTG	60	1'30	468
HPX	Hemopexin precursor	DS	I	20,806,896-20,824,484	1kbp from gene	GTTCAGCCAAAATCCCTGGGACT	GAAGAGAAGTGCCTTGCGTCTGA	60	2'00	671
HSC70	Heat shock cognate 70 kDa	TR	VII	11,121,002-11,134,155	5kbp from gene	CTGGCGAAACAGGAAAGGGA	CCACTTGCGTCGCTCCTC	60	1'30	413
HSP25	Heat shock protein 25 kDa	TR	II	19,711,740-19,749,566	Within gene	CGGACGCTTTTCCTTTGTTGCC	GCAGGGAAGTGAAGGCCAC	60	1'30	777
HSP47a	Heat shock protein 47 kDa subtype 1	TR	VII	12,192,868-12,215,855	Within gene	GATGAAAGGATGGTCGGCGTA	ACGGGGCAATGATCGTCAATG	58	1'00	2031
HSP70Aa	Heat shock protein 70 kDa isoform alpha subtype 1	TR	XI	12,697,367-12,699,286	10kbp from gene	GCACTACAGCTAAACGATACTAG	TCTGACAGGATGGGATGGC	58	1'30	800
HSP70Ab	Heat shock protein 70 kDa isoform alpha subtype 2	TR	I	10,989,308-10,994,114	5kbp from gene	AACACACTGGCGATCTACAG	TCTGTTTACTCCGGTCAATGA	53	1'30	291
HSP70Ac	Heat shock protein 70 kDa isoform alpha subtype 3	TR	XX	8,889,725-8,906,204	Within gene	AGCTTTACCTTCTGCCACAGGTA	GAGATGAGCTCCAGGTGGTCA	58	1'30	227
HSP70B	Heat shock protein 70 kDa isoform beta	TR	XII	10,121,285-10,123,204	10kbp from gene	GAGCACTAGTGGTGTCTGG	AGGTGTCCCTGATGCTATTATCC	60	2'00	606
HSP90Aa	Heat shock protein 90 kDa alpha subtype 1	TR	XVIII	14,758,907-14,763,738	3kbp from gene	GGCTATCCCATCACACTCTTTG	TTGGTGAGACTCTTGTAGAACTC	60	1'30	414
HSP90Ab	Heat shock protein 90 kDa alpha subtype 2	TR	XVIII	15,800,515-15,805,764	Within gene	ATCATGTAGCCCATGGTGGAG	GAGCCCATTGACGAGTACTG	58	1'00	751
HSP90B	Heat shock protein 90 kDa beta	TR	XIX	11,734,035-11,756,823	Within gene	TCTTCATTCAGTGCTTCACAGTTCA	CTGGTCAGGAAACATGGAGAG	60	2'00	899
IGF-I	Insulin-like growth factor I	GR	IV	32,094,000-32,108,128	0.2kbp from gene	CAAATGCTGCCCCAGCTGTTTCC	CAAACTGCAGCGTGTCGACCAG	56	1'30	1751
					7kbp from gene	GAAGGGCTTCAAGTACAAGGACAC	TGCAGACGTTCTCCTGGTGCTC	60	1'30	536
					7kbp from gene	ACCAATACGACATGAACCGGCAC	GTGTCCTTGTACTTGAAGCCCTTC	60	1'30	484
IGF-II	Insulin-like growth factor II	GR	XIX	13,287,163-13,294,000	2kbp from gene	GTTTCTGGTACCGTGACATTCCTG	GGCTCATTACGCAAGATACAGCTC	60	1'30	542
Kir2.1a	Kir2.1 channel subtype 1	OR	XI	8,871,806-8,881,805	8kbp from gene	CAGTTGGGTTCTGATAAGACAAGA	TTACAACTGACAGAGTCTGGTACTC	54	1'00	1910
Kir2.1c	Kir2.1 channel subtype 3	OR	XI	4,197,921-4,202,291	3kbp from gene	TATTAGGGTGATTTGGGGGCTC	CAGTGGTGCATTACTGCCT	58	1'30	300
Kir2.2	Kir2.2 channel	OR	V	10,617,610-10,627,609	5kbp from gene	AACCCACTCCATCATCTTCAG	CTGCAAATGGCAGCTTCTGTG	60	1'30	570
MSTNa	Myostatin subtype 1	GR	XVI	6,341,546-6,343,791	Within gene	CAGCTGTGGGTGAATCTGCGC	CTTCACCTCCATGAACGGTTGCTG	58	1'00	1092
MSTNb	Myostatin subtype 2	GR	I	27,825,752-27,827,140	Within gene	CAGGCGCCCAACATCAGCC	CTTGGTGGCCATGGTGATGATGGT	58	1'30	153
MYHa	Myosin heavy chain subtype 1	TR	XIX	2,500,192-2,512,408	8kbp from gene	AACAGTGAGGTGGCTCAGTGGAG	CGCTTCATTGTCTCCAGCTGATCCA	58	1'00	812
MYHe	Myosin heavy chain subtype 5	TR	XXI	2,918,630-2,940,444	10kbp from gene	GGGCCTTGACAAACAGGTTA	CCATCGGACAGGAAGAAAC	56	1'30	2858
NHE3	Sodium/hydrogen exchanger isoform 3	OR	X	2,085,482-2,100,701	3kbp from gene	CCAGCAGCTCAGGAGGAGT	GAGAAGCCGTTTGCTCTGTTTGG	60	1'30	766
NKCC1a	$Na^+/K^+/2Cl^-$ cotransporter isoform 1 subtype 1	OR	IX	13,973,744-13,991,096	Within gene	TTCAGCTCCGTGTTGCACTGCTC	GACACCAAGGAGCTGGAGAAGATG	56	1'30	1537
NKCC1b	$Na^+/K^+/2Cl^-$ cotransporter isoform 1 subtype 2	OR	XIII	19,857,060-19,870,757	Within gene	AGATGTTCAGCATGCAGCG	TTCGGCTGGGTCAAAGGAG	60	1'30	143
NPY2Rb	Neuropeptide Y receptor Y2 subtype 2	GR	IX	5,496,808-5,499,287	3kbp from gene	GTCACAATGGGACGAGTGCCTG	ATCGACAGCGACGGTGAGC	58	1'30	541
NPY7R	Neuropeptide Y receptor Y7	GR	IV	12,945,038-12,950,365	6kbp from gene	TGCTACATTATATTACTGGGCGGA	TCCAGCATGGACAAAGCCTG	60	2'00	1097
NPYP	Neuropeptide Y precursor	GR	X	9,524,349-9,538,081	Within gene	CTGCAGCGGACGGGATTAG	GTGGGTGATGATGATGGAGGA	56	1'30	466
PITX1	Paired-like homeodomain transcription factor 1	MR		AY517634.1*	Within gene	GTTTCACTTACCGAGAGCCCCG	AGGTTGGTCCACACGGCGATC	58	1'30	1001
PKMa	Pyruvate kinase subtype 1	TR	XIX	7,699,049-7,706,639	8kbp from gene	TCCAGGAGGCAGTACCAAGAAAAGG	GGCCATCTGTGAAACCTGCC	60	1'30	1131
PKMb	Pyruvate kinase subtype 2	TR	II	16,530,234-16,543,473	2kbp from gene	AGCAGCAATGCAGTCAGCTGC	GAGGATGGAGACTCGGATGC	56	1'30	388
PVALBb	Parvalbumin subtype 2	GR	XI	11,249,725-11,258,830	1kbp from gene	AGTGACACCTGACCTCAGCT	GCTGTAATAGTCAGGTTCTCCCTG	60	1'30	546
SHH	Sonic hedgehoc like protein	TR	XXI	8,991,593-9,005,094	Within gene	TGGCGAAGACCGCCGACATC	CACATAGAGGTTTCTGCACCTGAG	60	1'30	800
SLC14	Solute carrier family 14	OR	XIII	9,578,711-9,602,554	Within gene	CTCTGCCAGAGTCTATGGAACCTG	GTGCCATACAGCTGGAAGCCATC	60	1'30	1353
SSR1b	Somatostatin receptor subtype 1 type b	GR	XI	9,502,877-9,505,999	4kbp from gene	CTGCTTTGTTTCCATGGCAGCAC	GCACTGAGCTACTTCTTGGCA	60	2'00	520
T1R3	Taste receptor type 1 member 3 precursor	TS	XVII	6,411,674-6,415,316	4kbp from gene	ACCAGCATGTCCAGGTTGTCC	AAGTGCAGCGAGAGGAGTG	60	1'30	1662
TAAR	Amine-associated receptor	TS	XVII	864,777-877,400	Within gene	TGGCCAGTCAGCTGGACAA	GATTTCTTCTTGATGTCCGCT	53	1'30	141
TBX4	T-box4	MR	I	18,569,315-18,583,747	Within gene	TTCTCTGGTTAATGTCCAAAGGC	CTGCAGAGATGCTGCAGGA	53	1'30	1063
TTP	Tristetraprolin	DS	I	7,086,897-7,087,907	0.5kbp from gene	ACCAAAGGAATACTGCGGTGT	TGGAGGACAGCTACAGCAG	56	1'30	960

Gene function: DS, disease; GR, growth; MR, morphology; OR, osmoregulation; TR, thermal response; TS, taste. LG, linkage group; T_a, annealing temperature. * GenBank accession no.
