# Supplementary material for: Utility of sequenced genomes for microsatellite marker development in non-model organisms: a case study of functionally important genes in nine-spined sticklebacks (Pungitius pungitius)
Source: BMC Genomics. 2010 May 27;11:334. doi: 10.1186/1471-2164-11-334 (PMC2891615; doi:10.1186/1471-2164-11-334)
Supplement: Additional file 4 — Homology of nine-spined stickleback sequences in the three-spined stickleback genome and comparative genomic location of SSRs in these species. [file 1471-2164-11-334-S4.PDF]

Additional file 4: Homology of nine-spined stickleback sequences in the three-spined stickleback genome and comparative genomic location of SSRs in these species

Gene ID	Sequence homology in three-spined stickleback genome							SSR repeat motif (three-spined / nine-spined sticklebacks)					
	PCR (bp)	Primer	SEQ (bp)	LG	Position (bp)	E-value	ID (%)	Location 1	Location 2	Location 3	Location 4	Location 5	Location 6
ACAPRa	850	F & R	845	III	13,148,033-13,148,130	1.2E-61	93.9	$(CTC)_{10}$ / $(CTC)_{12}$	$(CTC)_{6}$ / $(CTC)_{4}$				
ACAPRb	700	F & R	714	VIII	4,749,465-4,749,726	4.0E-133	91.8	$(AC)_{17}$ / -	- / $(AACT)_{5}$				
AE1	800	F & R	745	XI	7,085,091-7,085,165	1.7E-91	97.3	$(TCG)_{4}$ / $(TCG)_{5}$	$(TCA)_{6}$ / $(TCG)_{7}$	$(CTC)_{6}$ / $(CTC)_{4}$	$(TCC)_{4}$ / -	$(GA)_{7}$ / $(GA)_{14}$	- / C_{15}
AQP9	1300	F	488	II	11,323,471-11,323,797	3.7E-128	90.9	$(TGT)_{7}$ / $(TGT)_{6}$					
		R	453	II	11,324,141-11,324,355	3.6E-95	90.1	- / T_{10}	T_{11} / -				
ATP1A1	1000	F & R	950	I	21,703,712-21,703,830	4.2E-119	90.8	$(TG)_{27}$ / $(TG)_{6}$	$(TG)_{5}$ / $(TG)_{6}$	- / $(CA)_{5}$			
ATP1A2	700	F & R	773	III	15,259,291-15,259,360	2.1E-36	99.8	$(GC)_{5}$ / $(TG)_{8}$	$(TG)_{10}$ / -	$(TG)_{8}$ / $(TG)_{5}$	$(GT)_{6}$ / $(GT)_{12}$		
ATP4A	300	F & R	290	XX	10,308,407-10,308,559	7.3E-77	87.0	$(TC)_{18}$ / $(TC)_{5}$					
ATP6V1Aa	700	F & R	720	XXI	7,742,630-7,742,781	3.0E-84	98.0	$(TG)_{11}$ / $(TG)_{6}$	$(TG)_{5}$ / $(TG)_{10}$				
ATP6V1Ab	200	F & R	194	VII	22,254,472-22,254,603	2.5E-55	94.7	$(CA)_{11}$ / $(CA)_{6}$					
CASR	1500	F & R	1485	VII	22,321,864-22,322,022	7.2E-115	93.1						
CFTR	700	F & R	466	XIX	10,187,080-10,187,173	1.5E-35	87.2	$(AC)_{40}$ / $(AC)_{27}$	$(CA)_{6}$ / $(CA)_{13}$	- / $(CA)_{5}$			
CLCN3	900	F & R	810	VII	2,431,938-2,432,049	7.1E-83	95.5	$(GT)_{9}$ / $(GT)_{8}$	T_{10} / -	$(CA)_{11}$ / $(CA)_{11}$			
CLCN4	400	F & R	383	I	27,638,297-27,638,507	9.6E-161	96.2	$(CTC)_{21}$ / $(CTC)_{5}$					
CLCN7	450	F & R	421	XI	14,735,892-14,735,988	1.9E-95	97.9	$(CAT)_{10}$ / $(CAT)_{8}$	$(CCA)_{4}$ / $(CCA)_{4}$	- / A_{10}	A_{13} / -	A_{11} / $(TG)_{10}$	
CLCNK	450	F & R	456	XII	5,805,198-5,805,361	5.7E-85	92.1	G_{12} / -	$(TA)_{8}$ / -				
CSP1	400	F & R	408	XI	4,640,549-4,640,758	7.0E-114	88.5	$(CA)_{13}$ / -					
CSP2	350	F & R	327	VII	4,341,487-4,341,606	4.3E-67	93.3	$(AAAT)_{5}$ / A_{11}					
DIO1	200	F & R	208	VIII	15,371,720-15,371,785	5.1E-46	100.0	$(TCAC)_{7}$ / $(TC)_{9}$					
eEF1A1b	400	F & R	381	IX	9,900,942-9,900,963	7.3E-04	100.0	$(AC)_{14}$ / $(AC)_{9}$					
FERH1	400	F & R	402	II	8,641,934-8,642,128	3.3E-95	90.8	$(TCG)_{4}$ / $(TCG)_{4}$	$(TCA)_{5}$ / -	$(CTC)_{5}$ / $(CTC)_{4}$	$(ATC)_{4}$ / $(ATC)_{5}$		
FGF2	1500	F	374	IV	3,336,218-3,336,334	1.6E-60	90.8						
		R	684	IV	3,337,047-3,337,112	9.4E-25	91.0	G_{12} / -					
FGF6a	700	F & R	595	XIX	19,488,280-19,488,426	1.2E-142	95.3	$(CT)_{40}$ / -					
FGF18	900	F & R	846	IV	10,297,300-10,297,630	0.0	96.7	$(AC)_{6}$ / $(AC)_{5}$	$(AG)_{10}$ / $(AG)_{12}$				
GH	900	F & R	861	XI	16,070,208-16,070,329	1.2E-61	89.4						
GHRH	400	F & R	325	XXI	9,523,999-9,524,127	4.5E-56	87.0	$(AT)_{8}$ / $(AT)_{8}$	A_{11} / A_{16}				
GHR-1	1100	F & R	997	XIII	5,672,730-5,672,979	7.3E-125	87.8	$(GT)_{5}$ / -					
	500	F & R	493	XIII	5,691,359-5,691,498	2.6E-122	92.3	- / $(AC)_{7}$	$(CA)_{13}$ / $(CA)_{7}$				
GHR-2	800	F & R	623	XIV	10,397,312-10,397,412	2.1E-56	91.2	- / $(TA)_{6}$					
GR1	700	F & R	690	IX	11,867,156-11,867,576	6.6E-208	90.3	$(TG)_{10}$ / -					
GR2	300	F & R	281	VII	23,192,194-23,192,382	1.2E-108	95.3	$(TGA)_{5}$ / -					
GTF2B	500	F & R	590	III	8,938,555-8,938,803	1.0E-158	92.4	$(CA)_{26}$ / -					
HPX	600	F & R	571	I	20,825,911-20,825,995	7.9E-68	100.0	- / $(CT)_{10}$	$(CA)_{5}$ / $(CA)_{11}$	$(AC)_{7}$ / $(AC)_{26}$	- / G_{12}		
HSC70	400	F & R	398	VII	11,115,820-11,116,058	9.8E-122	94.1	T_{20} / -					
HSP25	800	F & R	695	II	19,745,085-19,745,167	4.6E-41	96.5	$(AC)_{11}$ / $(AC)_{16}$	$(AC)_{33}$ / $(AC)_{13}$	- / C_{22}			
HSP47a	2000	F	493	VII	12,211,790-12,212,259	2.5E-218	95.3						
		R	218	VII	12,213,539-12,213,754	3.8E-96	95.4	- / $(CA)_{6}$					
HSP70Aa	800	F & R	769	XI	12,709,078-12,709,504	1.5E-264	92.6	$(CA)_{5}$ / -	$(AC)_{20}$ / $(AC)_{15}$				
HSP70Ab	300	F & R	230	I	10,999,484-10,999,568	3.4E-47	94.2	$(AC)_{12}$ / $(AC)_{7}$	$(ACGC)_{9}$ / -				
HSP70Ac	200	F & R	229	XX	8,893,407-8,893,528	7.4E-69	93.7	$(TG)_{16}$ / $(TG)_{5}$					
HSP70B	600	F & R	554	XII	10,111,209-10,111,364	2.1E-147	94.9	$(TC)_{13}$ / $(TC)_{14}$					
HSP90Aa	400	F & R	390	XVIII	14,766,036-14,766,233	1.9E-105	93.9	$(GAG)_{6}$ / $(GAG)_{4}$	$(AGA)_{5}$ / -				
HSP90Ab	700	F & R	705	XVIII	15,801,889-15,802,088	1.1E-171	96.5	- / $(CA)_{5}$	$(AC)_{13}$ / -				
HSP90B	900	F & R	777	XIX	11,751,508-11,751,672	1.1E-137	97.0	- / $(TCG)_{4}$					
IGF-I	1300	F	838	IV	32,098,293-32,098,477	3.3E-241	98.9						
	550	F & R	524	IV	32,091,527-32,091,896	1.9E-239	96.0	$(CGC)_{4}$ / -					
	450	F & R	474	IV	32,090,900-32,091,221	1.5E-189	93.8	- / $(CGC)_{4}$					
IGF-II	500	F & R	420	XIX	13,292,914-13,293,115	5.7E-123	98.0	$(TA)_{22}$ / $(TA)_{13}$	$(TA)_{27}$ / $(TA)_{5}$				
Kir2.1a	1600	F & R	1491	XI	8,886,397-8,886,748	5.1E-168	86.4	$(TA)_{15}$ / $(TA)_{10}$					
Kir2.1c	300	F & R	265	XI	4,205,564-4,205,679	1.2E-87	94.0	$(TG)_{20}$ / $(TG)_{7}$					
Kir2.2	550	F & R	522	V	10,628,690-10,628,934	5.3E-94	93.5	$(TC)_{6}$ / $(TC)_{15}$	$(CT)_{7}$ / $(CT)_{8}$				
MSTNa	800	F & R	726	XVI	6,342,870-6,343,122	3.0E-223	95.7	$(CA)_{15}$ / $(CA)_{5}$					
MSTNb	150	F & R	163	I	27,825,933-27,826,019	4.5E-38	94.3	$(GGA)_{6}$ / $(GGA)_{8}$					
MYHa	800	R	507	XIX	2,520,598-2,520,852	6.4E-160	95.7						
MYHe	3000	F	695	XXI	2,948,100-2,948,186	1.3E-38	89.1	- / $(GTAA)_{4}$					
		R	889	XXI	2,949,898-2,950,079	8.2E-141	87.0						
NHE3	900	F & R	870	X	2,102,910-2,103,147	9.6E-172	91.3	$(AT)_{15}$ / -					
NKCC1a	1800	R	489	IX	13,976,774-13,976,924	4.9E-43	88.2						
NKCC1b	150	F & R	127	XIII	19,868,542-19,868,613	5.1E-19	91.7	$(CA)_{18}$ / $(CA)_{13}$					
NPY2Rb	500	R	321	IX	5,502,443-5,502,486	6.3E-05	90.9						
NPY7R	1100	F & R	1052	IV	12,956,508-12,956,856	7.7E-277	90.1	$(ATA)_{8}$ / $(ATA)_{4}$	T_{13} / -				
NPYP	450	F & R	428	X	9,537,343-9,537,533	5.5E-105	92.2	$(GT)_{32}$ / -					
PITX1	1000	F & R	819		AY517634.1	2.0E-59	96.0	$(CCCT)_{4}$ / -	$(ATA)_{8}$ / -				
PKMa	1000	F & R	955	XIX	7,691,428-7,691,675	2.8E-179	92.8	$(GA)_{5}$ / $(GA)_{5}$	A_{11} / G_{20}				
PKMb	350	F & R	378	II	16,545,664-16,545,803	1.2E-98	95.0	$(CA)_{7}$ / -					
PVALBb	600	F & R	557	XI	11,259,930-11,260,021	5.1E-56	91.3	$(CA)_{23}$ / $(CA)_{6}$					
SHH	800	F & R	668	XXI	8,997,931-8,998,191	9.9E-187	94.3	$(TGT)_{6}$ / -	C_{11} / T_{10}				
SLC14	1200	F	333	XIII	9,587,394-9,587,474	1.9E-62	91.6	$(TG)_{7}$ / $(TG)_{9}$					
		R	690	XIII	9,588,065-9,588,160	1.2E-81	97.9	$(TG)_{14}$ / $(TG)_{13}$					
SSR1b	500	F & R	471	XI	9,507,902-9,508,044	9.8E-84	91.1	- / $(TG)_{12}$	$(GT)_{12}$ / $(GT)_{13}$				
T1R3	1800	F	411	XVII	6,406,681-6,406,747	1.1E-26	92.5						
		R	833	XVII	6,407,751-6,407,945	1.8E-98	89.3	C_{13} / -	$(AT)_{36}$ / -				
TAAR	150	F & R	140	XVII	877,299-877,363	6.9E-29	93.9	$(AC)_{17}$ / $(AC)_{12}$					
TBX4	1000	F & R	858	I	18,583,223-18,583,436	1.9E-267	95.3	$(GT)_{8}$ / $(GT)_{8}$					
TTP	1000	F & R	863	I	7,086,581-7,086,683	2.4E-210	97.1	$(CA)_{7}$ / -	$(TGT)_{4}$ / $(TGT)_{4}$				

PCR, PCR product size; F, forward; R, reverse; SEQ, sequence; LG, linkage group; E-value, BLAST hit score; ID, identity by BLAST.
